# Human DNA Telomeres in Presence of Oxidative Lesions: The Crucial Role of Electrostatic Interactions on the Stability of Guanine Quadruplexes

**DOI:** 10.3390/antiox8090337

**Published:** 2019-08-22

**Authors:** Cecilia Hognon, Adrien Gebus, Giampaolo Barone, Antonio Monari

**Affiliations:** 1 Université de Lorraine, CNRS, LPCT UMR 7019, F54000 Nancy, France; 2 Department of Biological, Chenical and Pharmaceutical Sciences and Technologies, Università di Palermo, Viale delle Scienze, 90128 Palermo, Italy

**Keywords:** human DNA telomeres, oxidative lesions, guanine quadruplexes, all atom molecular dynamics

## Abstract

By using all atom molecular dynamics simulations, we studied the behavior of human DNA telomere sequences in guanine quadruplex (G4) conformation and in the presence of oxidative lesions, namely abasic sites. In particular, we evidenced that while removing one guanine base induces a significant alteration and destabilization of the involved leaflet, human telomere oligomers tend, in most cases, to maintain at least a partial quadruplex structure, eventually by replacing the empty site with undamaged guanines of different leaflets. This study shows that (i) the disruption of the quadruplex leaflets induces the release of at least one of the potassium cations embedded in the quadruplex channel and that (ii) the electrostatic interactions of the DNA sequence with the aforementioned cations are fundamental to the maintenance of the global quadruplex structure.

## 1. Introduction

DNA is the essential biological macrostructure responsible for the storing, replication, and transduction of the genetic information of living organisms. As such, maintaining its chemical structure is essential to avoid genome instability that can lead either to cell death or to the development of mutations. However, DNA is constantly exposed to a number of endogenous or exogeneous stress sources [1] that may trigger chemical reactions resulting in the modification of its structure, i.e., DNA damage or lesions [2]. As a non-exhaustive list of stress sources, one can cite reactive oxygen species (ROS) produced by the cellular metabolism or by external agents [3,4] as well as ionization radiation [5], pollutants [6], or the exposure to UV light [7,8]. Different stress agents produce specific classes of lesions that may involve DNA nucleobases or the phosphate and sugar backbone components. The accumulation of DNA lesions, due to the imbalance between their production and repair, is not only related to the emergence of seriously debilitating diseases, such as cancers [9,10] or neurodegeneration [11,12,13], but also to the acceleration of the senescence process of cells and organisms [14].

Due to their oxidation potential guanine nucleobases represent hotspots for oxidative lesions that mainly result in the production of the widely studied 8-oxo-guanine (8oxoG) [15,16,17] or in the excision of the nucleobase to give rise to the so-called abasic or apurinic/apyrimidinic (AP) site [18,19,20], which is also produced as an intermediate of base excision repair (BER) processes [21,22,23,24,25]. Thymine oxidative lesions, such as formyl-uracyl [26], have been proven to act as trojan horses, inducing the further formation of thymine-dimers by photosensitizations [27,28]. As far as AP sites are concerned, their structural dynamics have been shown to be rather complex, especially in the case of cluster lesions, i.e., when AP sites are accumulated in close spatial proximity. Indeed, the coupling of the different AP sites in clustered lesions has been shown to significantly alter the B-DNA structural parameters [29] as well as the recognition of lesioned DNA by human and bacterial endonuclease and hence their repair efficiencies [30].

Some single-stranded DNA regions, characterized by a high density of guanine, such as the telomeric regions, are known to give rise to non-canonical arrangements such as the so-called guanine quadruplexes (G4s) [31,32,33,34,35,36]. Indeed, in G4s, guanines are organized in planes composed of four bases, also called a tetrad, further stabilized by the formation of Hoogsteen hydrogen bond networks. Tetrads can, in addition, form a π-stacking interaction with other planes, leading to a super structure composed of the superposition of different tetrads, up to three or four units. Interestingly, the specific arrangements dictated by the Hoogsteen hydrogen bonds [37,38,39,40] lead to an accumulation of negative charge close to the center of the plan, hence G4s need to be stabilized by the inclusion of cations, such as K^+^ and Na^+^, in their central channel [41]. G4s may exist in a delicate equilibrium between different conformers differing for the orientation of the dihedral angles of the backbone, giving rise to parallel, antiparallel, and hybrid arrangements [42]. In addition, the formation and stabilization of G4s induces the inhibition of the telomerase activity and hence may regulate the cells’ senescence. As such, some therapeutic strategies involving the stabilization of G4s by selective drugs have been proposed and actively studied as an alternative to conventional cancer chemotherapy [43,44,45,46] and more recently have been envisioned as antiviral agents—in particular against the Zika virus [47].

As G4s are formed in the region of DNA rich in guanine, they should be considered as obvious hotspots for the formation of lesions in conditions of oxidative stress. However, the interplay between oxidative lesions and the stability of G4s has been scarcely studied, despite the relevant biological role of G4s in the regulation of both gene expression and senescence. In this study, we focused on the human telomere (h-telo) sequence that is highly conserved among vertebrates and is known to form a parallel G4 arrangement. Specifically, we investigated the effects of the inclusion of one AP lesion at different positions of the DNA sequence. In this paper, we clearly show how the presence of lesions induces a partial disruption of the G4 structure that is, however, partially compensated by the tendency of the oligomer to adapt to the new conditions to avoid a total denaturation of the ordered arrangements. We also highlight the crucial role played by the G4′s stabilizing cations in maintaining the overall structure.

## 2. Materials and Methods

**Figure 1:**
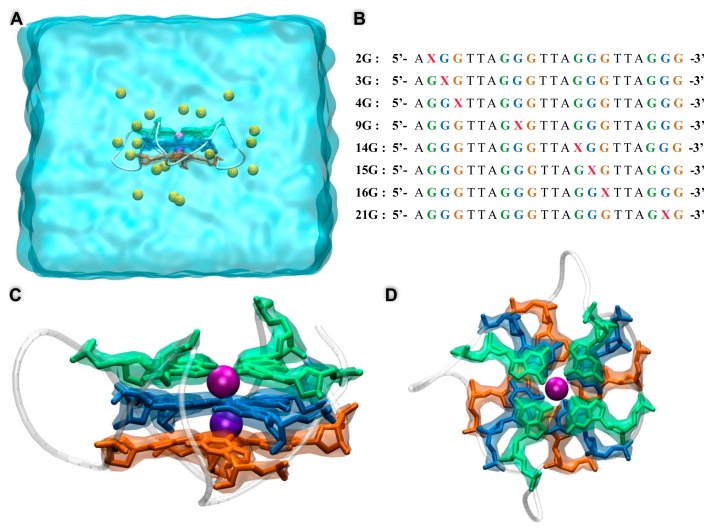
(**A**) Representation of the native human telomere (h-telo) guanine quadruplex (G4) sequence and the simulation box—each of the bases belonging to one of the G4 tetrads has been colored accordingly. (**B**) Representation of the lesioned sequence constructed for this work—note that **X** defines the AP site and the color code used is the same as in (A) allowing the easy identification of the tetrad to which the substituted guanine belonged. Side (**C**) and top (**D**) view of the native h-telo structure.

As a starting point of our simulations, we considered the h-telo structure provided by Neidle et al. (pdb code 1kf1) [48], comprising a 22 nucleobase sequence arranged in a parallel G4 structure comprised of three superposed tetrads. Even though this structure represents a very minimal model of the complex human telomere arrangement, whose single-strand sequence is much longer, its study is nonetheless crucial since the crystallographic structure of the G4 is available and because it represents a fundamental building block of the global telomere architecture. Indeed, studying the full overhanging single-stranded telomere would be prohibitive for all atom simulations and should require parameterization of coarse-grained or even mesoscopic models. As illustrated in Figure 1 and starting from the wild-type crystal structure, we built 8 replicas of h-telo by including an AP site on a specific position of the sequence and involving different tetrads. To avoid tackling a combinatorial problem, we reduced the number of replicas by taking into account only one lesion per replica. In addition, we shifted the lesion from one tetrad to the following one on the same position. We also considered two additional lesions involving either the central plane or the terminal guanine. Even though our model did not cover all the possible lesion arrangements, it represented, nonetheless, a reasonable exploration of the chemical space spanned by damage production. For readers’ convenience in this paper, the specific sequence containing a G4 lesion will be named as **XG** where **X** will be the position, counted from the 5′ end, of the guanine that has been substituted with an abasic site. All the G4 sequences were solvated in a cubic periodic water box of 60 Å, and K^+^ cations were added to ensure electroneutrality. Note that the K^+^ cations present in the pdb structure and located inside the G4 channel were conserved. DNA was modeled by the amber force field including the *bsc1* corrections [49,50], while water was described at the TIP3P level [51]. In the case of the AP site, we consistently used the same parameters as the ones obtained in our previous work [29,52] and built the replicas, using the standard amber procedure based on the restrained electrostatic potential (RESP) [53,54] protocol. All the systems were minimized for 1000 steps to remove bad contacts and then equilibrated and thermalized for 6 ns. For each of the strands, i.e., the native G4 and all the AP-containing sequences, all atom molecular dynamics (MD) simulations of 200 ns were performed in the constant pressure and temperature ensemble (NPT) at 300 K and 1 atm, using the NAMD code [55], while the trajectories were visualized and the results analyzed using VMD [56]. In all cases, the masses of non-water hydrogens were rescaled with the hydrogen mass repartition (HMR) procedure [57] allowing the use of a time-step of 4 fs in combination with the Rattle and Shake algorithms [58]. A cut-off for the electrostatic interactions of 10 Å was used for all the dynamics, while the particle mesh Ewald procedure (PME) [59] was consistently used to calculate electrostatic interactions. Finally, the harmonic constraints of 10 kcal/mol on the distance between the K^+^ and the center of mass of the most external tetrad were imposed via the Colvar module [60], and additional dynamics with the same protocol as the previous ones were realized.

## 3. Results

### 3.1. Equilibrium All Atom Dynamics

**Figure 2:**
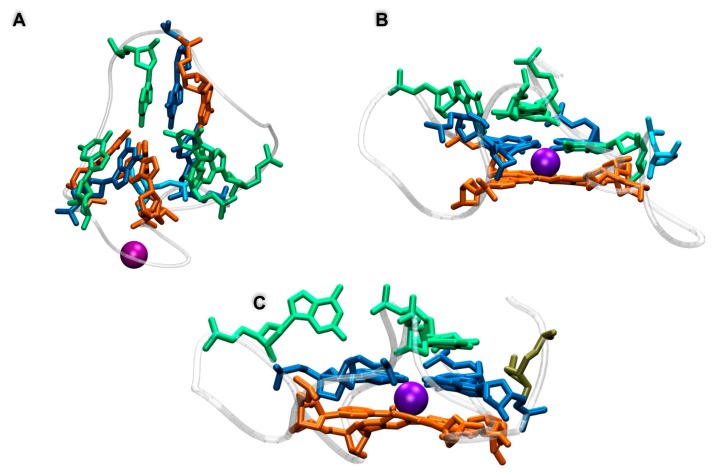
Representative snapshots illustrating the behavior of the different classes of G4: (**A**) total disruption of the G4 structure as experienced by **3G**, (**B**) substitution of the damaged nucleobase on the central tetrad as experienced by **9G**, and (**C**) disruption of the peripheral tetrad as evidenced by **2G**. The representative snapshots for the other damages are given in Supplementary Informations (SI).

As reported in Table 1 and Figure 2, the outcome of the MD simulations for the AP-containing strands is strongly dependent on the position of the lesion and, in particular, on the presence of the damage on the two external tetrads or on the central one.

**Figure 3:**
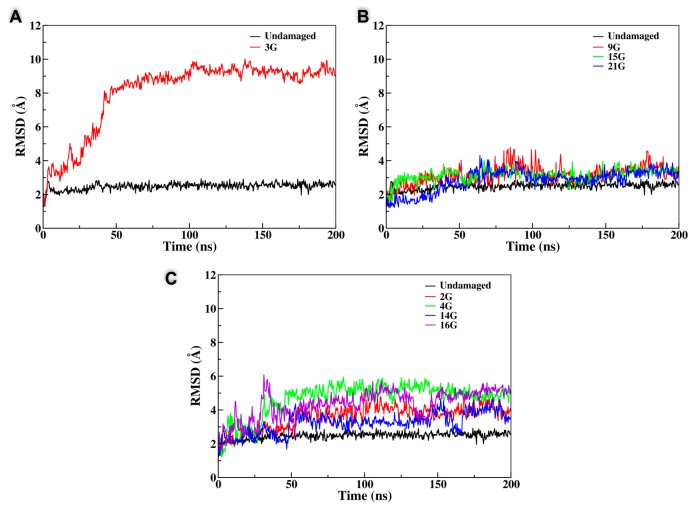
Time series of the root mean square deviation (RMSD) for all the different classes of G4, leading to the total destructuration of the G4 (**A**), to the substitution of the AP site in the central tetrad (**B**), and to the destructuration of the peripheral plane (**C**). The RMSD of the undamaged G4 is also reported for comparison.

**Figure 4:**
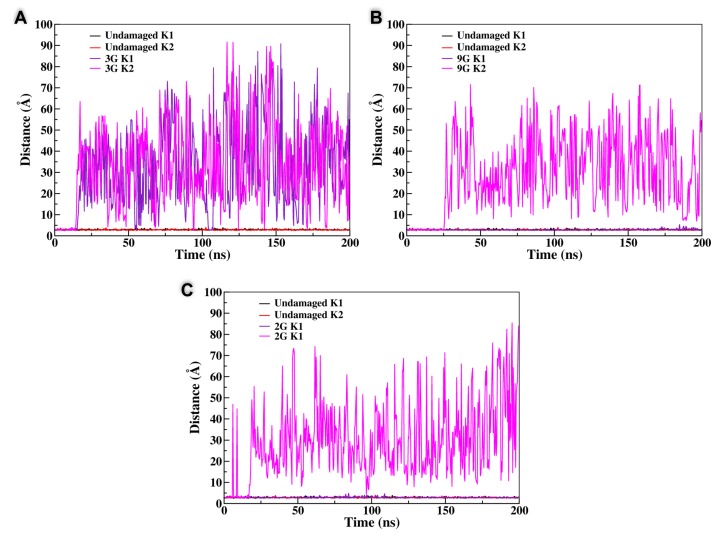
Time evolution of the distance between the central K^+^ and the undamaged guanine oxygen atom for the **3G** (**A**), **9G** (**B**), and **2G** (**C**) lesions, respectively. The time evolution of the distance for the undamaged quadruplex is also reported for comparison.

In the case of a lesion involving the peripheral planes, the behavior observed through the MD simulation was generally quite straightforward. After equilibration, and also due to the disruption of the Hoogsteen hydrogen bond network, the tetrad containing the AP site was rapidly disrupted and its structure was globally lost, even if in some cases the residual guanines remained partially locked by π-stacking interactions with the nearby tetrad. In addition to the pictorial representation provided in Figure 2, this structural transition can also be easily evidenced by the analysis of the time series of the root mean square deviation (RMSD) reported in Figure 3 that, although remaining quite small, generally presents an increase appearing at around 40 ns. Interestingly, the destructuration of the AP-containing tetrad was also accompanied by the release of one of the K^+^ cations, since the interaction with the remaining central plane was not sufficient to contrast the favorable release due to entropic and enthalpic reasons (see Figure 4). In contrast, the remaining two tetrads did not experience any significant distortion and remained stable throughout the simulations, ensuring a partial maintenance of the G4 arrangement. Coherently, the second K^+^ cation was persistently kept in the G4 channel, as evidenced by the distances reported in Figure 4. A partial difference, as reported in the SI, was observed for the **4G** sequence, in which, after an initial behavior coherent with the one previously described, we observed the insertion of one of the residual guanines of the disrupted tetrad into the central plane followed by the insertion of the expelled guanine to the third tetrad. Hence, and despite a more pronounced restructuration compared to the one experienced by the other members of this class, even the **4G** oligomer maintained two stable tetrads.

The situation was instead much more complex when the behavior of the central tetrad was analyzed. Indeed, for one arrangement, namely the **3G** strand, we observed the total disruption of the G4 conformation with the unfolding of all the three planes in about 30–40 ns, after which the system behaved like an unstructured DNA single strand; this was also accompanied by the important increase in the RMSD value, as shown in Figure 3. Not surprisingly, the destructuration of the whole G4 was also accompanied by the release in the bulk of both the K^+^ cations. However, the behavior of the other arrangements (**9G**, **15G**, and **21G**) was totally different and did not lead to the destructuration of the whole G4 structure. Indeed, in all three cases, one can observe that the AP on the central plane was substituted by one guanine coming from one of the terminal tetrads. This transition, that was accompanied by only a very modest increase of the RMSD, as highlighted in Figure 3, led inevitably to the disruption of the terminal tetrad from which the guanine base was taken and consequently to the release of one of the K^+^ cations. However, filling the gap produced by the AP site in the central tetrad, and in contrast with the situation observed for the **3G** case, had the advantage of maintaining the stability of two over three tetrads and hence kept a partial stability of the G4 arrangement. Partially counterintuitively, the RMSD values achieved for all the G4s following this trend were even lower than the one obtained in the case of the unfolding of the terminal tetrads, again pointing to the fact that the stabilization of the central tetrad will be strongly beneficial in assuring the persistence of the global G4 arrangement. Interestingly, it was evident that the substitution of the AP site in the central leaflet by one of the external guanines was also coupled with a non-negligible reorganization of the G4 external loops, and in particular with the inversion of some of the backbone dihedrals. However, this transition was rapid and did not exceed 40–50 ns and hence was not accompanied by significant free-energy barriers. This fact could also be a consequence of the inherent propensity of G4 to coexist in a polymorphic ensemble leading to the well-known parallel, antiparallel, and hybrid arrangements and to their interconversion depending on the environmental conditions [61].

### 3.2. Constrained K^+^ Dynamics

**Figure 5:**
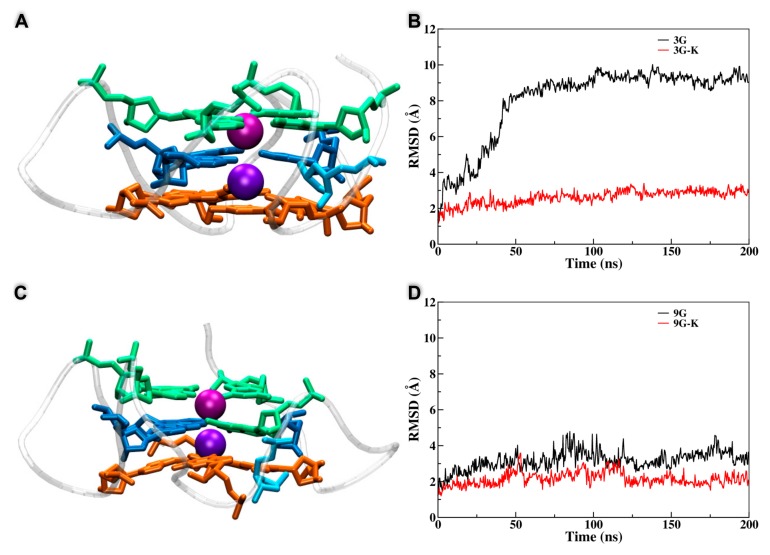
Representative snapshots and time evolution of the RMSD for the dynamic of the **3G** (**A**,**B**) and **9G** (**C**,**D**) systems in which the positions of the K^+^ have been constrained. The RMSD values for the corresponding unconstrained dynamics are also reported for comparison.

From the different behavior observed previously, it is clear that on one hand the central plane plays a special role in assuring the stability of the G4 arrangement and on the other hand that the correlation between the stability of the tetrads and the release of the K^+^ cations is also fundamental. To disentangle these effects and provide a sound interpretation of the dynamics of the AP-containing G4, we performed two additional MD simulations in which the position of the central cations were constrained to avoid their diffusion in the bulk. In particular, we considered the case of the **3G** arrangement, leading to the total disruption of the quadruplex, and the **9G** oligomer that leads to the substitution of the damaged guanine in the central tetrad. The results for the constrained and unconstrained dynamics are collected in Figure 5 and clearly show that, compared with the equilibrium simulations, the situation was totally altered. In particular, the **3G** strand was now extremely stable and all of its tetrads remained well structured, as was also confirmed by the small value of the RMSD (Figure 5B). The same was true for the **9G** case in which, and because of the constraints exerted on the cation position, the substitution of the oxidized guanine in the central tetrad did not take place anymore, while all the tetrads maintained their stable G4 conformation. Hence, it is evident that the interplay between the G4 stability and the maintenance of the interactions with the potassium cations residing in the central channel is fundamental to determine the outcome of their structural evolution and the strategies taken to counterbalance the perturbation brought by the oxidative lesions.

## 4. Discussion and Conclusions

A large part of the DNA sequence in humans, vertebrates, and other organisms is non-coding and hence is not devoted to information transduction [62]. However, non-coding DNA areas still exert fundamental biological roles, most notably the so-called promoting regions are related to the regulation of gene expressions [63,64]. Non-coding DNA is also involved in the regulation of the cells’ cycle and of their longevity, in particular via the telomeric regions of DNA. Eukaryotic telomeres protect the edge of chromosomes and consist of a rather conserved double-strand structure, with an overhanging, much shorter, single strand that may organize in G4 [35]. The length of the telomeres is progressively shortened with the number of cell replications [65,66,67] and regulated by the action of the telomerase. Telomerase is a ribonucleoprotein that is able to maintain the length of telomeres by catalyzing an addition to the edge of the DNA strand of a specific sequence (TTAGGG) in vertebrates, using the constituent RNA as a template [68]. The progressive shortening of the terminal sequences allows a critical limit to be reached in the telomere’s length triggering cell senescence and its death; the deregulation of the telomere’s modulation, for instance by telomerase overexpression, may lead to a sort of immortalization of the cells that is at the base of cancer invasiveness [69,70], and, as such, G4s are emerging as promising targets for novel chemotherapeutic strategies [45,71].

Our results, obtained for damaged G4 DNA, clearly allow us to outline general tendencies concerning the behavior of such systems in the presence of AP sites. Indeed, when a lesion is present on one of the peripheral planes, its outcome is quite obvious and leads to the disruption of the involved tetrad, while the rest of the G4 structure remains almost unaltered. On the other hand, the disruption of the edge planes also leads to the release of one of the central cations. This is not entirely surprising since, and because of its position, the K^+^ cations are known to develop strong electrostatic interactions with the two tetrads among which they are intercalated. However, such a situation reaches a critical point when the AP site involves the central tetrad, i.e., the one that is neighboring both K^+^ ions. From our results, it is evident that this central plane plays a peculiar role and in case of its disruption, as observed for the **3G** sequence, the full G4 is destabilized and destroyed while the cations are rapidly released in the solution bulk. However, in the majority of the cases considered, and probably thanks to the inherent flexibility of the G4 loops as opposed to their rigid core, when in presence of a lesion in its central plane, the oligomers prefer to sacrifice one of the external tetrads using one of the undamaged guanine bases to fill the gap in the central plane, hence ensuring the stability of the latter and the disruption of the former. In turn, such an evolution also allows the interaction to be maintained with at least one of the K^+^ cations and hence the global structure of the two remaining planes. The crucial role of the electrostatic interaction with the central cation is also evidenced by the fact that, when their position is kept constrained, hence forbidding their release in the bulk, the structure of the G4 is kept intact and no base substitution takes place. In the future, we plan to confirm our modeling and simulation results via in vitro experiments in which the AP site can be precisely inserted in the same oligomers as studied in the present contribution while their structural determination could be inferred either via electronic circular dichroism, which has proven a very sensitive technique to unravel the structural modification of DNA and G4 in particular [72,73,74] and offers a one-to-one mapping with experience [42]. In the same spirit, the effects of salt concentration or nature, and the presence of a crowding environment, can also be envisaged.

All in all, it appears that, while the electrostatic interactions are crucial, the flexibility of the G4 loops is also instrumental in the tendency shown by G4s to maintain structural stability and a quadruplex arrangement, even in the presence of lesions such as AP. Indeed, consistently, the G4 will prefer sacrificing one of the undamaged external guanine tetrads to reinstate the stability in the central plane, hence avoiding the whole structural destabilization. Even though this aspect should be confirmed by experimental investigations, important hypotheses concerning the biological relevance of our results can already be settled. Indeed, the concentration of APs will tend to increase in critical conditions for the cell, and particularly in the case of an important oxidative stress, which can be correlated with genomic instability and mutations. In such conditions, the tendency to maintain, at least partially, the G4 structure can be seen as extremely positive since it will avoid the overactivation of the telomerase and hence the deregulation of the senescence processes, also limiting the possibility to confer the cell’s immortality properties that may ultimately favor carcinogenesis when combined with a high possibility of mutations.

AP sites are an important class of lesions that can also result as intermediates of the repair of other oxidative DNA damage. As such, in the future, we plan to extend our study to the analysis of the behavior of G4 in the presence of other lesions, such as 8oxoG, or eventually cluster lesions featuring the close proximity of AP and 8oxoG. In addition, the study of the interaction of damaged h-telo sequences with human telomerase and eventually with the RNA template used by the inverse polymerase will also contribute to shedding further light on the regulation of the senescence in conditions of high oxidative stress, while also allowing the proposition of novel chemotherapeutic agents or radiotherapy sensitizers for cancer treatments. However, to offer a quantitative determination of the binding free-energy profile, such studies will require advanced sampling MD techniques, such as extended adaptative biased force [75] or its combination with metadynamics [76]; hence they will require an important computational effort and the definition of stable structures in the space phase and of proper collective variables. In this respect, the results of the present contribution are also important in providing an understanding of the unbiased dynamic of oxidatively damaged G4s.

## Figures and Tables

**Table 1:** Evolution of the G4 structure as a function of the position of the apurinic/apyrimidinic (AP) site.

Sequence	Disruption of the Peripheral Tetrad	Substitution of the Central Tetrad	Total Disruption of the G4 Structure
**2G**	x	-	-
**3G**	-	-	x
**4G**	x	x	-
**9G**	-	x	-
**14G**	x	-	-
**15G**	-	x	-
**16G**	x	-	-
**21G**	-	x	-

## Supplementary Materials

The following are available online at https://www.mdpi.com/2076-3921/8/9/337/s1: Figure S1: Representative snapshots of all the G4 evolutions, Figure S2: Representative snapshots of the **4G** structure, and Figure S3: Time evolution of the distances between K^+^ and the guanine for all the damaged quadruplexes.
