# Supplementary material for: Human DNA Telomeres in Presence of Oxidative Lesions: The Crucial Role of Electrostatic Interactions on the Stability of Guanine Quadruplexes
Source: Antioxidants (Basel). 2019 Aug 22;8(9):337. doi: 10.3390/antiox8090337 (PMC6770428; doi:10.3390/antiox8090337)
Supplement: Supplementary file 1 [file antioxidants-08-00337-s001.pdf]

Supporting Information:

Human DNA Telomeres in Presence of Oxidative Lesions: The Crucial Role of Electrostatic Interactions on the Stability of Guanine Quadruplexes

Cecilia Hognon [1,*], Adrien Gebus [1], Giampaolo Barone [2] and Antonio Monari [1,*]

[1] Université de Lorraine, CNRS, LPCT UMR 7019, F54000 Nancy, France

[2] Department of Biological, Chenical and Pharmaceutical Sciences and Technologies, Università di Palermo, Viale delle Scienze, 90128 Palermo, Italy

* Correspondence: cecilia.hognon@univ-lorraine.fr (C.H.); antonio.monari@univ-lorraine.fr (A.M.)

Received: 15 July 2019; Accepted: 20 August 2019; Published: date

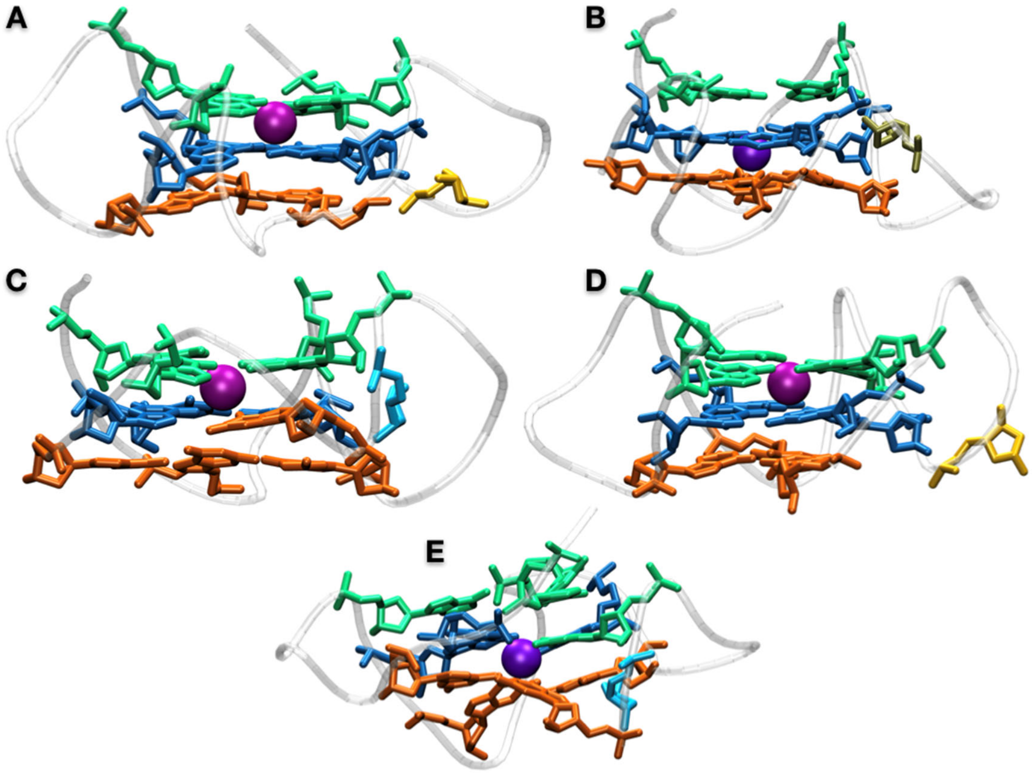

Figure 1. Representative snapshots of all the G4 evolution **4G** (**A**), **14G** (**B**), **15G** (**C**), **16G** (**D**), **21G** (**E**).

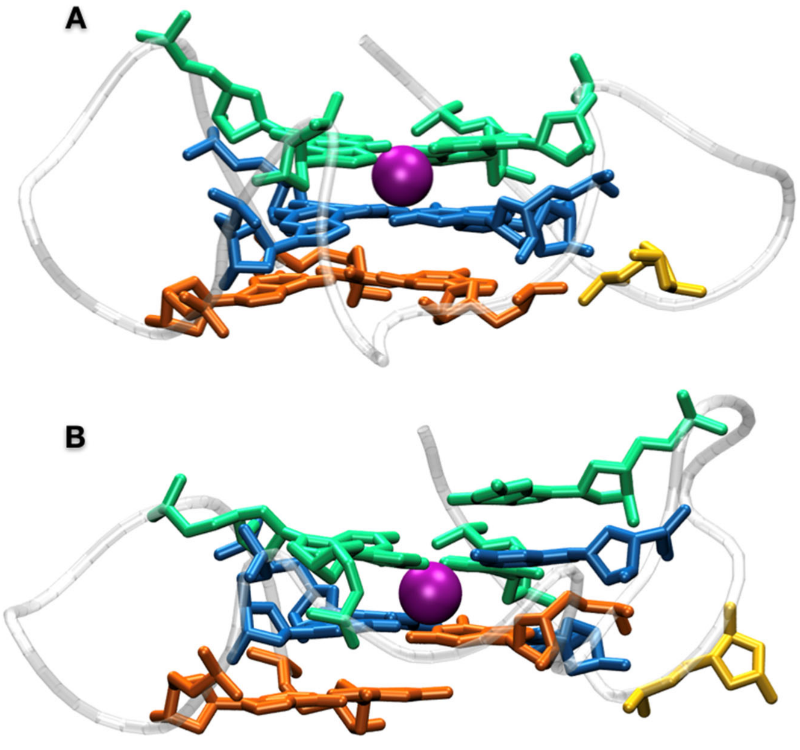

Figure S2: Representative snapshots for the **4G** quadruplex before (**A**) and after (**B**) substitution of one of the guanine of the peripheral tetrad into the central one. Note that the substitution takes place after 30ns and is then persisting until the end of the simulation (i.e., for about 60 ns).

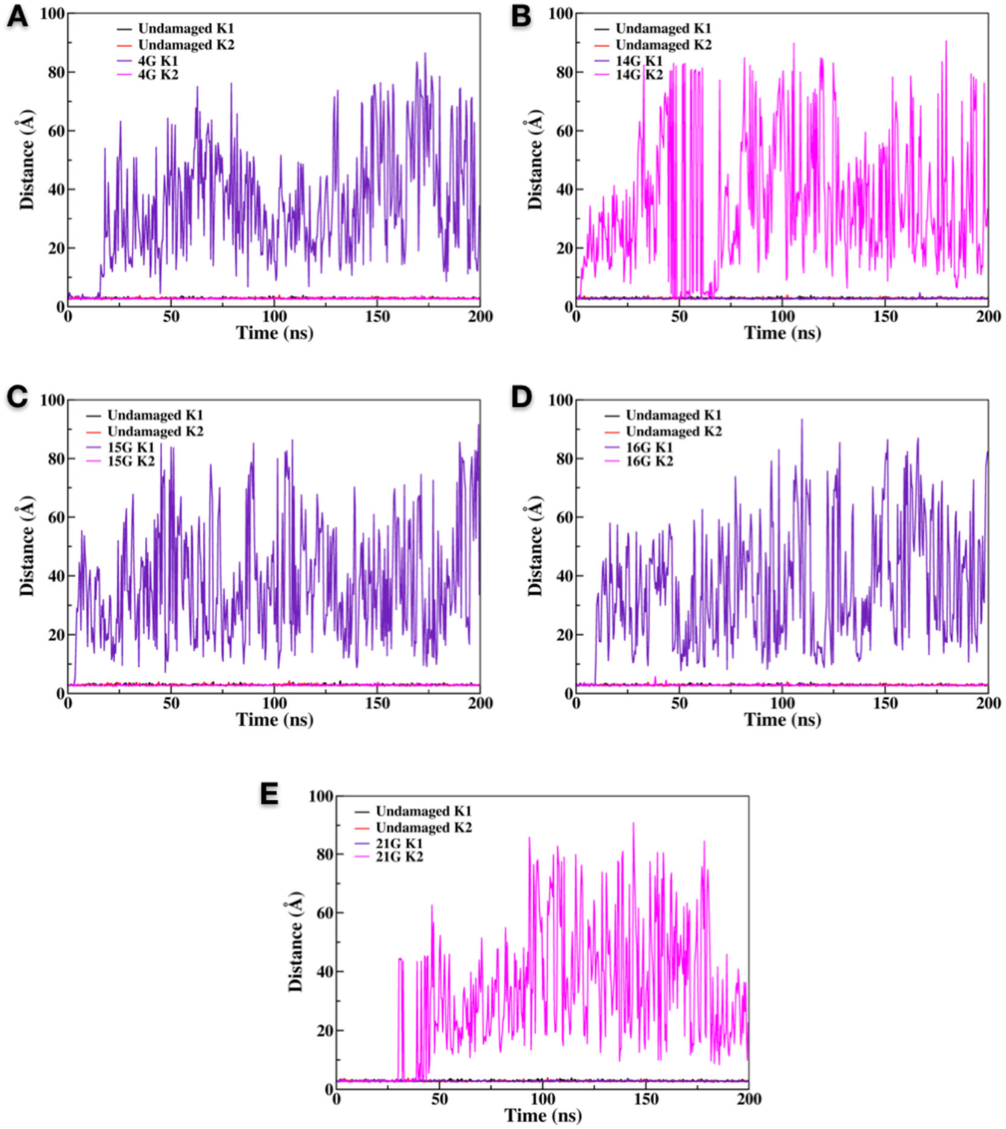

Figure S3: Time evolution of the distances between K^+ and the tetrad guanine for all the damaged quadruplexes **4G** (**A**), **14G** (**B**), **15G** (**C**), **16G** (**D**), **21G** (**E**).
